# MRI Evaluation of Complete and Near-Complete Response after Neoadjuvant Therapy in Patients with Locally Advanced Rectal Cancer

**DOI:** 10.3390/diagnostics12040921

**Published:** 2022-04-07

**Authors:** Anca-Raluca Popita, Cosmin Lisencu, Adriana Rusu, Cristian Popita, Calin Cainap, Alexandru Irimie, Liliana Resiga, Alina Munteanu, Zsolt Fekete, Radu Badea

**Affiliations:** 1 “Ion Chiricuţă” Oncology Institute, 400015 Cluj-Napoca, Romania; ralucapopita@gmail.com (A.-R.P.); cosminlisencu@yahoo.com (C.L.); cristianpopita@gmail.com (C.P.); airimie@umfcluj.ro (A.I.); liliana.resiga@gmail.com (L.R.); muntean.alina@yahoo.fr (A.M.); fekete.zsolt@umfcluj.ro (Z.F.); 2 Medical Imaging Department, “Iuliu Haţieganu” University of Medicine and Pharmacy, 400162 Cluj-Napoca, Romania; rbadea2012@gmail.com; 3 Oncology Department, “Iuliu Haţieganu” University of Medicine and Pharmacy, 400015 Cluj-Napoca, Romania; 4 Diabetes and Nutrition Diseases Department, “Iuliu Haţieganu” University of Medicine and Pharmacy, 400006 Cluj-Napoca, Romania; adriana.rusu@umfcluj.ro

**Keywords:** locally advanced rectal cancers, neoadjuvant treatment, restaging MRI, complete response, near-complete response

## Abstract

**Purpose** To evaluate MRI performance in restaging locally advanced rectal cancers (LARC) after neoadjuvant chemoradiotherapy (nCRT) and interobserver agreement in identifying complete response (CR) and near-complete response (nCR). **Methods** 40 patients with CR and nCR on restaging MRI, surgery and/or endoscopy were enrolled. Two radiologists independently scored the restaging MRI and reported the presence of split scar sign (SSS) and MRI tumor regression grade (mrTRG). Diagnostic accuracy and ROC curves were calculated for single and combined sequences, with inter-reader agreement. **Results** Diagnostic performance was good for detecting CR and weaker for nCR. T2WI had the highest AUCs among individual sequences. There was a significant positive correlation between SSS and CR, with high Sp (89.5%/73.7%) and PPV (90%/79.2%) for both Readers. Similar accuracy rates were observed for the combination of sequences, with AUCs of 0.828–0.847 for CR and 0.690–0.762 for nCR. Interobserver agreement was strong for SSS, moderate for T2WI, weak for the combination of sequences. **Conclusions** Restaging MRI had good diagnostic performance in identifying CR and nCR. SSS had high Sp and PPV in diagnosing CR, with a strong level of interobserver agreement. T2WI with DWI was the optimal combination of sequences for selecting good responders.

## 1. Introduction

The main role of neoadjuvant chemoradiation therapy (nCRT) in locally advanced rectal cancers (LARC) is to downsize and downstage the primary tumor, yielding a pathologic complete response in 10% to 30% of patients [1,2]. Although radical surgery remains the standard of treatment according to actual guidelines [3,4], in the last two decades a ”watch-and-wait” strategy has been increasingly offered as an option for patients with a good response to nCRT [5]. This is due to the substantial postoperative morbidity of rectal resections [6]. Even though it is still controversial, the trend of conservative treatment with observation alone is sustained by the favorable oncologic outcome in regard to recurrence and survival [1].

The goal of the “watch-and-wait” approach is to identify patients with clinical complete response after neoadjuvant therapy and enter them in a strict follow-up protocol, including digital rectal exam (DRE), endoscopy and magnetic resonance imaging (MRI), with salvage surgery in case of tumor recurrence [5,7,8]. These complementary examinations should be used together for a better evaluation of tumor response.

While DRE and endoscopy are used in evaluating the luminal complete response, the restaging MRI provides additional information in detecting residual tumor in the layers of the rectal wall and the mesorectum, response at the level of adenopathy, as well as in reporting tumor regrowth [9]. Morphologic T2-weighted imaging (T2WI) is agreed to be a key sequence in the evaluation of rectal tumor response after nCRT, also showing the post-therapeutic changes (fibrosis, desmoplastic reaction, edema or mucin production) [10,11]. The use of functional sequences—such as diffusion-weighted imaging (DWI) and dynamic contrast-enhanced (DCE) MRI—can provide additional information about tumor cellularity and perfusion characteristics, improving the post-therapeutic assessment [9,12]. A major challenge currently remains to increase the accuracy of MRI in diagnosing complete response [13].

Over the last years, the concept of near-complete response (nCR) has emerged as a third form of response at restaging, besides complete response (CR) and partial response (PR). Identifying this category is important because several studies have demonstrated that a significant proportion of near-complete responders may convert into complete responders at reassessment, secondary to prolonged time to act of treatment, especially after radiotherapy [14].

Radiologists should know the role of MRI in detecting CR and nCR of rectal cancer after treatment, understanding its benefits, strengths, limitations and new trends. To promote global standardization for rectal cancer imaging, the European Society of Gastrointestinal and Abdominal Radiology (ESGAR) and the Society of Abdominal Radiology (SAR) proposed consensus recommendations and structured report templates for the MRI assessment [10,11]. Structured reports for primary staging and restaging rectal cancer have also been developed by several national radiological societies [15,16,17].

The aim of this study was to evaluate the performance of different morphologic and functional MRI sequences in restaging patients with LARC after nCRT and to assess the interobserver agreement in identifying CR and nCR.

## 2. Materials and Methods

### 2.1. Study Design and Patients

This was a prospective, observational, single-center study performed between March 2017 and December 2021 in the Oncological Institute Cluj-Napoca. Consecutive patients with histologically confirmed rectal tumors, locally advanced, treated with nCRT were enrolled. Rectal MRI was performed before and after nCRT. Patients with CR or nCR on either restaging MRI or pathological reports from surgery and/or endoscopy were included in this study.

We excluded complete or near-complete responders at endoscopy and/or surgery who did not have restaging MRI, non-oncologic deaths and patients who were not followed up at least 6 months after nCRT.

The follow-up protocol consisted of DRE, MRI and endoscopy performed every three months during the first year, every six months in the second year, and annually up to five years after inclusion—protocol after Maas et al. [13].

The study was approved by the Ethics Commission of the “Iuliu Hatieganu” University of Medicine and Pharmacy Cluj-Napoca, Nr. 336, dated 30 September 2014, and written informed consent was obtained from all patients prior to any study procedures. The study was performed in agreement with The Code of Ethics of the World Medical Association (Helsinki Declaration) for experiments involving human subjects.

### 2.2. MRI Protocol

MRI examinations were performed using a 1.5T Magnetom Aera scanner (Siemens Healthcare, Erlangen, Germany), with a 30-element body matrix coil (acquisition parameters are shown in Table 1). For all staging and restaging assessments, we used a protocol derived from the recommendations for MRI rectal cancer evaluation and reporting provided by the ESGAR guidelines [10]. Patients were examined in feet-first supine position, using the same MRI protocol: T2WI, DWI with apparent diffusion coefficient (ADC) map, T2 high-resolution (T2HR) angulated perpendicular and parallel to the rectal tumor axis, and T1C (Table 1). A microenema prior to MRI exam was used in a subset of patients. DCE included axial T1 sequences (36 phases; mean acquisition time: 5 min) with gadolinium-based contrast agent Gadobutrol (Gadovist, Bayer) administered i.v. as a bolus, at a dose of 0.1 mmol/kg body weight at a rate of 2 mL/s, followed by a 20 mL saline flush. 

The timeframes of performing the MRI were at baseline (before any treatment procedure) and at restaging, with a median time between the finalization of radiotherapy and MRI reassessment of 6.5 weeks.

### 2.3. Image Interpretation 

All MRI examinations—both the baseline and the restaging assessments—were read by two independent radiologists (Readers 1 and 2) with 9 and 8 years of experience in rectal MRI, who were blinded to each other’s readings. The images were analyzed using Syngo (VB17) software, commercially available applications and OsiriX MD viewer. Both radiologists were required to independently read the restaging MRI, report the T stage, N stage, the presence of tumoral perirectal deposits (N1c), extramural vascular invasion (EMVI) and circumferential resection margin (CRM). Five grade confidence level scores modified after Maas et al. [13], were used to evaluate response to treatment on T2WI, DWI and T1C, respectively (Table 2). 

We reported an MRI tumor regression grade (mrTRG) based on the combination of T2WI, DWI and T1C (Table 3), adjusted after the system developed by the Mercury study group [18,19,20]. The highest individual sequence score determined the overall score for the combination of sequences and mrTRG. For patients with mucinous tumors, we used the modified mrTRG system proposed by Park et al. [21]. Radiologists were asked to note the presence or absence of the split scar sign (SSS) on T2HR sequences, as described by Santiago et al. [22].

MRI findings were reported as CR, nCR or PR. Image interpretation with scoring was done before surgery and/or endoscopy, so the radiologists were blinded to pathological findings. 

### 2.4. Definition of Response and Reference Standards 

Tumor response was evaluated with restaging MRI and correlated with the pathological reports from surgery (total mesorectal excision—TME)—the reference standard for all operated patients. MRI was considered “true positive” for CR if the pathologist confirmed it (CR: ymrTRG1; pTRG0). MRI was “true positive” for nCR if the histopathology analysis confirmed the MRI report (nCR: ymrTRG2; pTRG1)—Table 3. Cases were counted as “true negative” if both radiologist and pathologist reported residual tumor (PR: ymrTRG2,3; pTRG2). 

Endoscopy, local recurrence-free follow-up (for at least 6 months) and biopsy (when available) were the reference standard for patients managed non-operatively. Endoscopy was suggestive of CR if a flat white scar with telangiectasia was seen at the tumor site, without ulcer or nodularity. If minor mucosal abnormality or shallow ulcer/red scar were present, endoscopy was indicative of nCR [14,23]. MRI was considered “true positive” for CR if endoscopy confirmed complete response (endoscopy CR) and “true positive” for nCR if endoscopy confirmed near-complete response (endoscopy nCR). “True negative” cases were counted if MRI and endoscopy found residual tumor (endoscopy PR). “True positive” cases for CR and nCR had no involved nodes on MRI, based on the criteria from the guidelines for nodal restaging [10] (p. 1470).

### 2.5. Histopathology Analysis

Surgically resected specimens from TME were thoroughly sectioned, with careful examination of the tumor site, and evaluated according to the guidelines of the American Joint Committee on Cancer, using the TNM staging system. A modified Ryan scheme (Table 3) for scoring tumor regression grade (pTRG) after neoadjuvant therapy was used [24]. 

**Figure 1:**
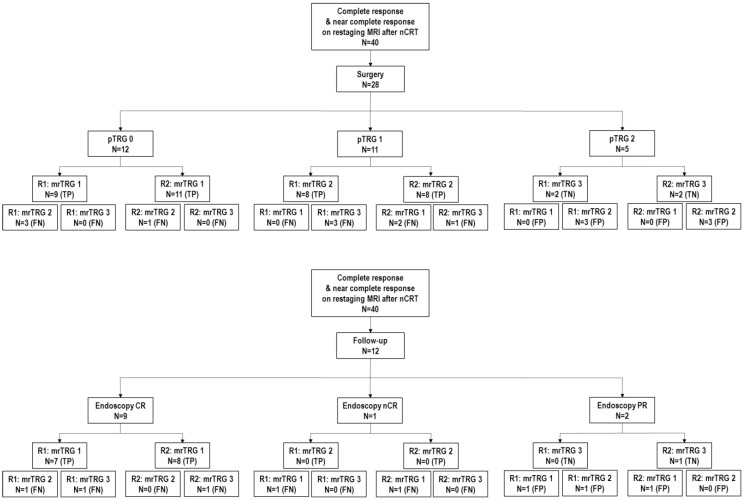
Flowchart of treatment and tumor assessments for included patients. nCRT = neoadjuvant chemoradiotherapy; pTRG = pathologic tumor regression grade; mrTRG = MRI tumor regression grade; TN = true negative; FP = false positive; TP = true positive; FN = false negative; CR = complete response; nCR = near-complete response; PR = partial response.

Patients were classified as being with CR (pTRG0), nCR (pTRG1) and PR (pTRG2) (Figure 1).

### 2.6. Statistical Analyses

Data were analyzed using MedCalc 20.026 (Ostend, Belgium: MedCalc Software Ltd.) and IBM SPSS Statistics version 26 (Armonk, NY, USA: IBM Corp.). Numerical variables were summarized using descriptive statistics: number and proportion for qualitative variables, mean and standard deviation or median (quartile 1; quartile 3) for continuous variables. Student t-test and independent samples median test (depending on its distribution) were used to compare numerical variables. 

The diagnostic performance of restaging MRI in detecting CR or nCR was assessed by receiver operating characteristics (ROC) curves, area under the curve (AUC), Se, Sp, positive predictive value (PPV) and negative predictive value (NPV) for individual and combined sequences, as well as for mrTRG, separately for both Readers. AUCs were compared between MRI sequences and mrTRG in MedCalc using the methodology of DeLong et al. [25]. The accuracy rate was calculated as Se×response in the sample + Sp × (1 − response in the sample), separately for CR and nCR, for both Readers. Interobserver agreement was evaluated by Interrater agreement Cohen’s Kappa coefficient. The correlation between SSS at restaging MRI and the type of response at endoscopy and/or surgery was assessed by Spearman correlation coefficients. A *p*-value < 0.05 was considered statistically significant.

## 3. Results

### 3.1. Description of the Study Sample

During the above-mentioned period, 120 consecutive patients with locally advanced rectal cancer were assessed by pelvic MRI, before and after nCRT. After completion of nCRT, 45 patients had CR or nCR on either restaging MRI, pathological reports from surgery or endoscopy. Five patients were excluded from this analysis: two did not perform restaging MRI before surgery, one was missed due to non-oncologic death and two patients were not followed-up for at least six months in the “watch-and-wait” protocol.

40 patients fulfilled the inclusion criteria and were enrolled in this study (23 men and 17 women; mean age 58.8 years; age range 25–80 years). Three patients had a mucinous type of rectal adenocarcinoma. Low rectal tumors (located < 6 cm from the anal verge) were present in 45% of cases (Table 4).

28 patients (70%) underwent surgery, with a median time between the end of nCRT and restaging MRI of 6.5 weeks and between restaging MRI and surgery of 3.5 weeks. Among the operated patients, 12 (42.8%) had CR, 11 (39%) had nCR and 5 (17.8%) had PR, according to the pathological modified Ryan score (Figure 1).

**Figure 2:**
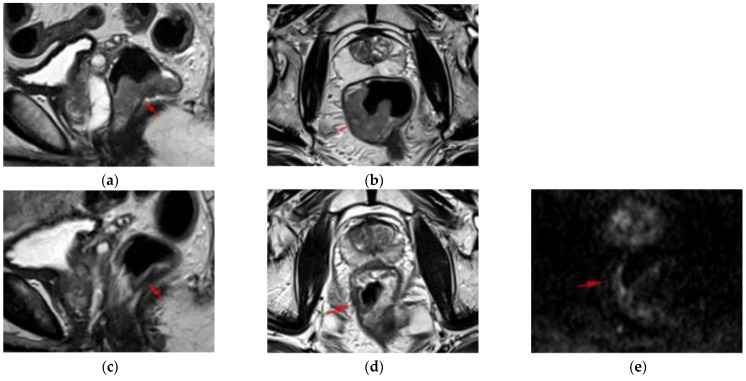

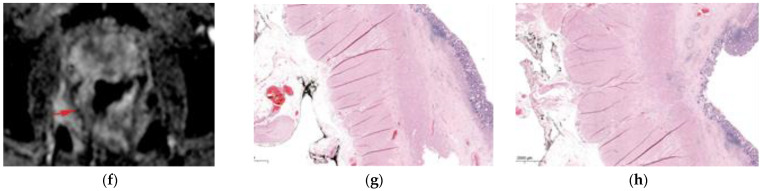
A 74-year-old male patient, initially staged mrLR3CRM+N+N1c-EMVI-, with a complete response at restaging MRI and histopathology (yp T0N0, modified Ryan score 0). (**a**,**b**) Staging MRI: sagittal and axial T2WI HR showing a tumor in the distal rectum mrLR3, on the right lateral side (red arrow). (**c**–**f**) Restaging MRI after nCRT: sagittal (**c**) and axial T2WI HR (**d**) showing marked reduction in tumor size with positive split scar sign (SSS+, red arrow), score 0 on T2WI; DWI score 0: linear high signal on the luminal side of the right lateral wall (**e**) also high on ADC (**f**), corresponding to the mucosa; (**g**,**h**) hematoxylin and eosin-stained 4-µm cut slices through the tumor scar illustrating the intact mucosa, dense fibrosis in the submucosa, thickened muscularis propria and fibrosis in the perirectal fat.

A patient with CR at restaging MRI, confirmed by the histopathological report from surgery, is presented in Figure 2.

**Figure 3:**
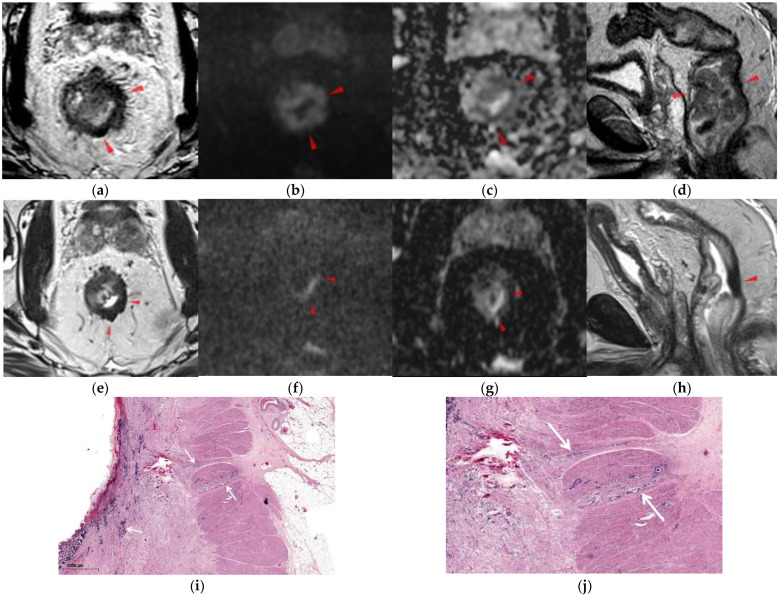
A 58-year-old male patient, initially staged T3cCRM-N+N1c-EMVI+, with near-complete response at restaging MRI and histopathology (yp T3N0, modified Ryan score 1). (**a**–**d**) Staging MRI showing low and middle rectal tumor (red arrowheads): axial T2WI HR (**a**), DWI (**b**), ADC (**c**), sagittal T2WI (**d**). (**e**–**h**) Restaging MRI after nCRT: axial T2WI HR (**e**), DWI (**f**), ADC (**g**) sagittal T2WI (**h**) showing marked tumor reduction with pronounced hypointense wall thickening between 2–8 o’clock, without isointense signal on T2WI (score 1, SSS-). The high signal on b1000 DW image (**f**), also high on ADC (**g**), was interpreted as T2 shine-through effects from fluid in the rectal lumen, indicating no clear areas of residual hyperintense signal on b ≥ 1000 (score 1). (**i**,**j**) hematoxylin and eosin-stained 4-µm cut slices through the tumor scar illustrate denudated mucosa, dense fibrosis in the submucosa, muscularis propria and in the perirectal fat, with rare small groups of cancer cells in muscularis propria and submucosa (white arrows).

Figure 3 illustrates a patient with nCR at MRI and histopathology after total mesorectal excision.

For 12 patients out of 40, the restaging MRI was correlated with endoscopy. Nine patients had CR (75%), one had nCR (8.3%) and two (16.6%) had residual tumor at endoscopy (Figure 1). Complete and near-complete responders entered the “watch-and-wait” protocol: six patients had sustained CR confirmed by DRE, MRI and endoscopy; one patient with initial CR at endoscopy had pathological nCR at six months surgery; two complete responders had tumor regrowth at six months MRI, confirmed by surgery; a patient with nCR at initial endoscopy became a sustained complete responder on the follow-up examinations.

**Figure 4:**
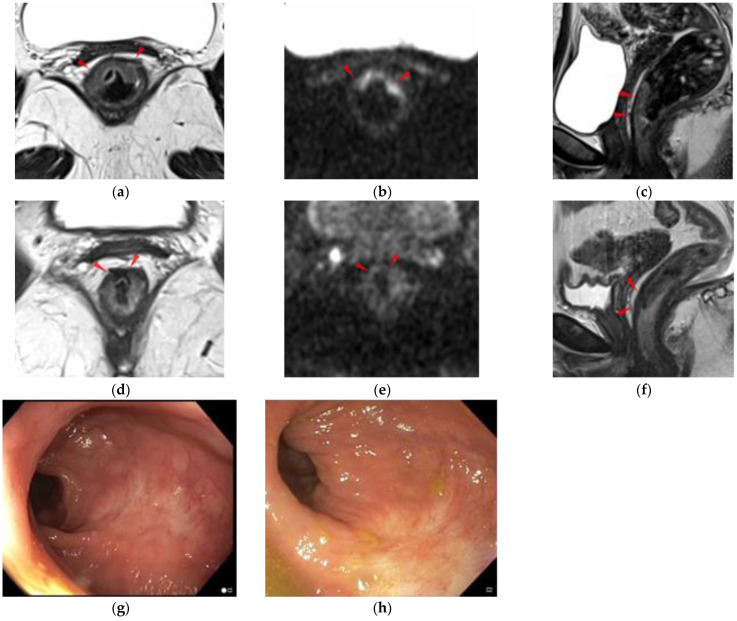
A 40-year-old female patient, initially staged mrLR3CRM+N+N1c+EMVI-, with a complete response at restaging MRI and endoscopy. (**a**–**c**) Staging MRI: axial T2WI HR (**a**), axial DWI (**b**), sagittal T2WI (**c**) showing a low rectal tumor (red arrowheads); (**d**–**f**) Restaging MRI after nCRT: axial (**d**) and sagittal T2WI HR (**f**) showing a pronounced reduction in size with positive split scar sign (SSS+, red arrowhead), score 0 on T2WI. No residual hyperintense signal on b ≥ 1000, with score 0 on DWI (**e**); (**g**,**h**) Flat white scar with telangiectasias at endoscopy, in accordance with clinical complete response.

A case with a complete response after nCRT, followed according to the “watch-and-wait” protocol, is illustrated in Figure 4.

### 3.2. Tumor Assessment Results by Readers

When data were analyzed overall, irrespective of the gold standard available at the restaging timepoint after nCRT, Reader 1 correctly identified 26 cases (65%)—24 “true positive” for CR and nCR (16 patients had surgery, 8 confirmed by endoscopy) and 2 “true negative” cases. Reader 2 correctly identified 30 cases (75%)—27 “true positive” for CR and nCR (19 patients had surgery, 8 confirmed by endoscopy) and 3 “true negative” (Figure 1). N1c, EMVI and CRM were negative in all restaging MRI examinations for both Readers, confirmed for all patients who underwent surgery. Reader 1 described residual adenopathy in two cases, one of these was also found in Reader 2′s report; the pathology report confirmed residual adenopathy (yN+).

### 3.3. Diagnostic Performance of MRI for Detecting Complete Response 

**Figure 5:**
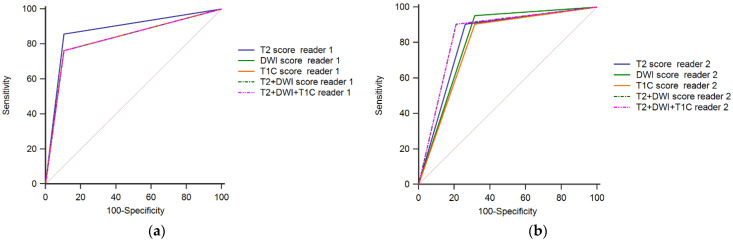
Receiver operating characteristics for the detection of complete response (Reader 1—panel (**a**); Reader 2—panel (**b**)).

Diagnostic performance was good for detecting CR. For Reader 1, the Sp was similar for all individual and combined sequences assessed (89.5%). Se, PPV, NPV, AUC and accuracy rates had the highest values for T2WI (85.7%, 90.0%, 85.0%, 0.876 and 87.5%, respectively). Similar AUCs were observed for DWI, T1C and for the combination of sequences analyzed (*p*-values for the difference between individual AUC were ≥0.05; Table 5; Figure 5). 

For Reader 2, the highest values for Se and NPV were observed for DWI (95.2%, 92.9%). The combination of sequences—T2WI+DWI and T2WI+DWI+T1C—had the highest Sp, PPV, AUCs and accuracy rates (79.0%, 82.6%, 0.847 and 85.0%, respectively), followed by T2WI score (73.7%, 79.2%, 0.821 and 82.5%). DWI had the highest Se and NPV among the sequences assessed. No significant differences for AUCs were observed between MRI sequences (*p*-value for the difference between individual AUC were > 0.05; Table 5, Figure 5).

The accuracy rates for mrTRG were 82.5% for Reader 1 and 85.0% for Reader 2, respectively.

The Sp and PPV of SSS for detecting CR were 89.5% and 90.0% for Reader 1, and 73.7% and 79.2% for Reader 2. Accuracy rates were 87.5% and 82.5%, respectively.

### 3.4. Diagnostic Performance of MRI for Detecting near-Complete Response 

**Figure 6:**
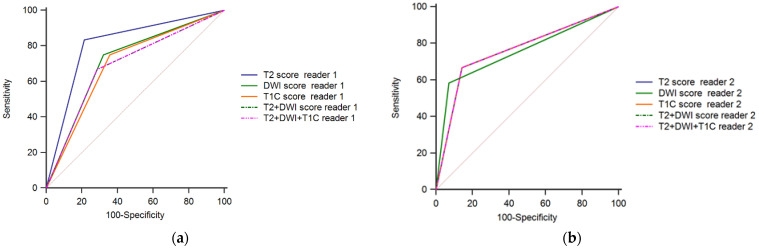
Receiver operating characteristics for the detection of near-complete response (Reader 1—panel (**a**); Reader 2—panel (**b**)).

Overall, the AUCs for both Readers were lower than those for detecting CR. For Reader 1, the highest Sp, PPV and accuracy rate were observed for T2WI (78.6%, 62.5% and 80.0%, respectively). AUC was significantly higher for T2WI than T1C, T2WI+DWI and T2WI+DWI+T1C (*p*-value for this comparison <0.05) (Table 6, Figure 6).

For Reader 2, DWI had the highest Sp, PPV and accuracy rate (92.9%, 77.8% and 82.5%, respectively), while Se was the lowest for this sequence. No significant differences for AUCs were observed between MRI sequences (*p*-values for the comparisons between individual AUCs >0.05 (Table 6, Figure 6).

The accuracy rates for mrTRG were 70.0% for Reader 1 and 80.0% for Reader 2, respectively.

### 3.5. Interobserver Agreement

Interobserver agreement was strong for SSS (k = 0.800), moderate for T2WI and weak for the combination of sequences. For identifying CR or nCR, the Kappa Cohen coefficients of agreement varied for different sequences assessed, ranging from 0.368—for DWI score, to 0.605—for T2WI score (Table 7).

## 4. Discussion

The focus of this study was to evaluate the performance of MRI sequences in diagnosing CR and nCR of LARC after nCRT, with surgery or endoscopy as the reference standard. 

The morphologic high-resolution T2WI sequences in three planes—sagittal and angulated perpendicular and parallel to the rectal tumor axis—were the mainstay of the protocol in assessing response. Scoring T2WI sequences relied on the visual appreciation of the relative proportion of low signal (corresponding to fibrosis) and intermediate signal intensity (residual tumor) within the treated neoplasia. 

We obtained high Se and Sp for both Readers when using T2WI score to identify CR, suggesting good diagnostic accuracy of this sequence. For detecting nCR, both Readers obtained good Sp, with good Se for Reader 1 and moderate Se for Reader 2 when using T2WI sequence. Similar to our results, recent studies from Nahas et al. (2019) and Ko et al. (2019) reported good Se (61% and 70.6%) and high Sp (89.6% and 95.3%) of T2WI for diagnosing a CR [26,27]. Nevertheless, a meta-analysis from 2020 including 17 studies showed a large heterogeneity of data, with a mild inverse relationship between Se and Sp of T2WI for diagnosing CR, the summary Se and Sp being 49% and 86%, respectively [28]. An explanation might be the various criteria used by different authors to diagnose CR on T2WI. Treatment-induced fibrosis and edema that mimic residual tumor also influenced the false negative rate, explaining the heterogeneous data reported. For the diagnosis of a nCR, a systematic review from van der Paardt et al. also reported good Sp and moderate Se, with mean values of 89.8% and 55.3% [29], similar to the ones we obtained in the current analysis. 

Interestingly, in our study, we obtained a very good Sp (89.5% Reader 1/73.7% Reader 2) and PPV (90% Reader 1/79.2% Reader 2) for predicting a CR, a high Se (85.7% Reader 1/90.5% Reader 2) in not missing complete responders and a strong level of agreement between Readers in the detection of CR using the SSS, with a Kappa Cohen coefficient of 0.800 (*p* < 0.001). The SSS has been described by Santiago et al. [22] as a characteristic morphologic pattern of the tumor scar, seen on high resolution T2WI sequence, having high Sp and PPV for identifying a CR. A positive SSS consists of an organized layered hypointense fibrosis at the tumor bed, which includes, besides mrTRG1 endoluminal scar, a perirectal layer of fibrosis separated by an intermediate signal intensity thickened, partially fibrotic muscularis propria [22,30]. The authors found that a positive sign at the restaging MRI had a very high Sp (97%) and PPV (93/94%) for a sustained CR, with a substantial interobserver agreement (k = 0.69; *p* < 0.01).

The diagnostic performance for identifying a CR was also good for both Readers when using DWI, with an AUC of 0.828 for Reader 1 and 0.818 for Reader 2. Nevertheless, a minimal level of agreement between Readers was achieved for the DWI score, with variability in interpreting qualitatively the presence or absence of hyperintense foci on high b-value at the site of the primary tumor, as shown by the values of Se and Sp of the two Readers. The lack of anatomical details and susceptibility artifacts on DWI can lead to interpretation errors and variability between Readers, as other authors previously reported [31]. Adding DWI to T2WI improved the diagnostic performance of MRI for Reader 2 in identifying the CR, but not for Reader 1, and improved the agreement among the Readers. Similar to our results for Reader 2, other studies reported AUCs between 0.77 and 0.85 when using a combined T2WI and DWI score for diagnosing CR [32] and better performance of these combined sequences with an improvement in diagnostic accuracy as compared to T2WI alone [28,33]. On the other hand, a meta-analysis of 14 studies from Wu et al. showed a non-significant increase in sensitivity for the prediction of tumor response when adding DWI to T2WI [34].

Diagnostic performance was weaker when using DWI and T2WI+DWI scores for detecting nCR than those obtained for CR, with lower AUCs for both Readers. No statistically significant differences between T2WI, DWI and T2WI+DWI AUCs were observed for Reader 2. Variable data were found in previous studies on DWI performance in detecting nCR. The variability in the diagnostic performance of this sequence is mainly due to the patterns of response to nCRT—fragmentation or shrinkage—and to cellular heterogeneity of the treated tumor, being inexact in differentiating between mere fibrosis, fibrosis containing vital tumor cells and small residual tumor [35].

Adding the T1C sequence to the standard MRI protocol (T2WI and DWI) showed no improvement in diagnostic accuracy for both Readers in identifying CR and nCR. These results are in accordance with previous studies from the literature that found no added value for qualitative assessment of DCE in selecting good responders [36]. 

MRI TRG is an imaging counterpart of TRG systems used in histopathology. Incorporating all sequence findings and using the highest score between sequences, we created a modified mrTRG system to grade fibrotic response. We compared mrTRG with pTRG and/or endoscopy. Our modified mrTRG had a good Sp and NPV, for both Readers, in diagnosing complete and near-complete responders. AUCs varied between 0.828–0.847 for CR and 0.690–0.762 for nCR. However, a weak interobserver agreement with a Kappa Cohen coefficient of 0.544 was observed. Other studies reported similar or higher Sp (88.7–94.2%) with lower sensitivity (16.7–33%) for complete responders. Se improved (56–60%) when CR and nCR were combined in selecting good responders [37]. The agreement between mrTRG graded on T2WI and pTRG is low in other literature published data, with limited performance to detect CR (Se 74%, Sp 63%) [20].

We acknowledge several limitations of our study. First, the number of patients is relatively small, being divided into patients who underwent surgery and a smaller subgroup of watch-and-wait patients. The time intervals, end of nCRT-restaging MRI and MRI-surgery, varied across the included patients and this might have influenced the results. The follow-up period was between 6 and 24 months for sustained complete responders so that there were complete responders with a short follow-up; this is important because local regrowth is generally reported to occur within the first 18 to 24 months [38]. We included in the study two patients with short-course radiotherapy with consolidation chemotherapy, who had no surgery and were followed according to the “watch-and-wait” protocol; pathological CR rates are known to be lower with this type of treatment, compared to long-course chemoradiation [39]. The use of a microenema prior to MRI could not be applied to all cases, and this could explain the potential pitfalls of the DWI sequence, caused by susceptibility artifacts. The MRI reports were based on the visual assessment of sequences and not on quantitative techniques, but this is similar to how a radiologist interprets cases in daily clinical practice. Our algorithm to combine T2WI, DWI and T1C data used the highest score for the overall assessment, and this might have influenced our results. 

## 5. Conclusions

Restaging MRI of LARC after nCRT had good diagnostic performance in identifying complete and near-complete responders. Using the SSS, both Readers obtained high Sp and PPV in diagnosing CR, with a strong level of inter-observer agreement. Dynamic contrast-enhanced MRI added to the standard protocol did not improve diagnostic accuracy. T2WI performed better than other individual sequences in diagnosing CR or nCR and the use of T2WI in three planes along with DWI was the optimal combination of sequences for selecting good responders. However, this is a single-center study including a small sample size; its results cannot be generalized and further studies with larger samples are needed to confirm our data.

## Figures and Tables

**Table 1:** Restaging pelvic MRI acquisition parameters.

Magnetom Aera 1.5-T	Sagital T2WI	Axial T2WI	Oblique Axial T2WI	Oblique Coronal T2WI	Axial DWI	Axial T1C (DCE)
Sequence	TSE	TSE	TSE	TSE	EPI DWI	VIBE
TR (ms)	5920	6380	5630	2670	6700	4.46
TE (ms)	108	114	108	108	75	1.72
ETL	17	17	17	17	-	5
FOV (mm^2^)	220	360	200	200	220	260
Flip angle (°)	160	160	160	160	-	12
Matrix	241 × 320	166 × 384	275 × 320	275 × 320	126 × 126	154 × 192
B-values	-	-	-	-	50, 500, 1000, 1500	-
Slice thickness (mm)	3	3	3	3	3	3.5
Gap (mm)	0	0.9	0	0	0	0.7

TR = repetition time; TE = echo time; ETL = echo train length; FOV = field of view; TSE = turbo spin echo; EPI = echo planar imaging; DCE = dynamic contrast-enhanced; VIBE = ultra-fast gradient echo. Oblique axial and coronal T2WI scans are oriented perpendicular and parallel to the rectal tumor axis, respectively.

**Table 2:** Definitions of confidence level scores for assessment of response at tumor location and mesorectum on different MRI sequences.

CL Score	T2WI	DWI	T1C	Response
0	Normalized rectal wall or linear/crescentic 1–2 mm hypointense scar at the mucosa-submucosa or positive SSS; no involved nodes	No residual hyperintense signal on b ≥ 1000 images with low ADC at former tumor location	Normalized rectal wall or progressive enhancement of the layered fibrotic wall; no involved nodes	Complete response
1	Pronounced hypointense wall thickening without isointense signal; no involved nodes	No clear areas of residual hyperintense signal on b ≥ 1000 images	Delayed or progressive contrast-enhanced wall thickening; no involved nodes	Near-complete response
2	Irregular wall thickening with both hypointense and isointense signal; no involved nodes	Possible foci of hyperintense signal on b ≥ 1000 images with low ADC in an area of irregular wall thickening	Irregular progressive contrast-enhanced wall thickening; no involved nodes	Near-complete response
3	Small residual isointense mass and/or involved nodes	Small but obvious area of hyperintense signal on b ≥ 1000 images with low ADC	Small early contrast-enhanced residual mass and/or involved nodes	Partial response
4	Gross residual isointense mass and/or involved nodes	Marked hyperintense signal at former tumor location on b ≥ 1000 images with low ADC	Gross early contrast-enhanced residual mass and/or involved nodes	Poor/no response

CL = confidence level; SSS = split scar sign.

**Table 3:** Modified MRI tumor regression grade and pathologic tumor regression grade with modified Ryan scheme.

SSS	T2WI Score	DWI Score	T1C Score	mrTRG	Response	pTRG	Pathology
+	0	0	0	1	Complete response	0	No viable cancer cells
-	1 2	1 2	1 2	2	Near-complete response	1	Single cells or rare small groups of cancer cells
-	3	3	3	3	Partial response	2	Residual cancer with evident tumor regression but more than single cells or rare small groups of cancer cells
-	4	4	4	4	Poor response	3	Extensive residual cancer with no evident tumor regression
-	5	5	5	5	No response		

mrTRG = modified MRI tumor regression grade; pTRG = pathologic tumor regression grade; SSS = split scar sign; + = present; - = absent.

**Table 4:** Characteristics of patients enrolled.

Parameters	Data
Gender, n (%) Male Female	23 (57.5) 17 (42.5)
Age (years), mean ± SD ≤49, n (%) 50–59, n (%) 60–69, n (%) ≥70, n (%)	58.8 ± 12.8 10 (25.0) 9 (22.5) 11 (27.5) 10 (25.0)
Tumor differentiation degree, n (%) G1 G2 G3	8 (20.0) 30 (75.0) 2 (5.0)
Tumor location from anal verge, n (%) <6 cm ≥6 cm	18 (45.0) 22 (55.0)
Neoadjuvant treatment Long-course chemoradiation, n (%) without consolidation CT with consolidation CT Short-course radiotherapy with consolidation CT, n (%)	38 (95.0) 30 8 2 (5.0)
Operated patients, n (%)	28 (70.0)
Patients on follow-up, n (%)	12 (30.0)
Time nCRT-to-restaging MRI (months), median (Q1; Q3)	6.5 (6.0; 8.5)
Time restaging MRI-to-surgery (months), median (Q1; Q3)	3.5 (2.0; 6.0)
Time nCRT-to-surgery (months), median (Q1; Q3)	11.5 (9.0; 14.5)

SD = standard deviation; n (%) = number (percentage) of patients; G = tumor differentiation degree; CT = chemotherapy; Q1 = quartile 1; Q3 = quartile.

**Table 5:** Diagnostic performance of restaging MRI according to Reader to correctly identify a complete response.

	Sensitivity, % (95%CI)	Specificity, % (95%CI)	PPV, % (95%CI)	NPV, % (95%CI)	AUC (95%CI)	Accuracy Rate, %
***Reader 1***						
T2WI score	85.7 (63.7; 97.0)	89.5 (66.9; 98.7)	90.0 (70.6; 97.1)	85.0 (66.3; 94.2)	0.876 (0.733; 0.959)	87.5
DWI score	76.2 (52.8; 91.8)	89.5 (66.9; 98.7)	88.9 (67.8; 96.8)	77.3 (60.9; 88.1)	0.828 (0.676; 0.929)	82.5
T1C score	76.2 (52.8; 91.8)	89.5 (66.9; 98.7)	88.9 (67.8; 96.8)	77.3 (60.9; 88.1)	0.828 (0.676; 0.929)	82.5
T2WI+DWI score	76.2 (52.8; 91.8)	89.5 (66.9; 98.7)	88.9 (67.8; 96.8)	77.3 (60.9; 88.1)	0.828 (0.676; 0.929)	82.5
T2WI+DWI+T1C score	76.2 (52.8; 91.8)	89.5 (66.9; 98.7)	88.9 (67.8; 96.8)	77.3 (60.9; 88.1)	0.828 (0.676; 0.929)	82.5
mrTRG	76.2 (52.8; 91.8)	89.5 (66.9; 98.7)	88.9 (67.8; 96.8)	77.3 (60.9; 88.1)	0.828 (0.676; 0.929)	82.5
SSS	85.7 (63.7; 97.0)	89.5 (66.9; 98.7)	90.0 (70.6; 97.1)	85.0 (66.3; 94.2)	0.876 (0.733; 0.959)	87.5
***Reader 2***						
T2WI score	90.5 (69.6; 98.8)	73.7 (48.8; 90.9)	79.2 (63.9; 89.1)	87.5 (64.6; 96.4)	0.821 (0.667; 0.924)	82.5
DWI score	95.2 (76.2; 99.9)	68.4 (43.4; 87.4)	76.9 (63.1; 86.7)	92.9 (65.2; 98.9)	0.818 (0.664; 0.922)	82.5
T1C score	90.5 (69.6; 98.8)	68.4 (43.4; 87.4)	76.0 (61.7; 86.2)	86.7 (62.7; 96.2)	0.794 (0.637; 0.906)	80.0
T2WI+DWI score	90.5 (69.6; 98.8)	79.0 (54.4; 93.9)	82.6 (66.3; 92.0)	88.2 (66.3; 96.6)	0.847 (0.698; 0.941)	85.0
T2WI+DWI+T1C score	90.5 (69.6; 98.8)	79.0 (54.4; 93.9)	82.6 (66.3; 92.0)	88.2 (66.3; 96.6)	0.847 (0.698; 0.941)	85.0
mrTRG	90.5 (69.6; 98.8)	79.0 (54.4; 93.9)	82.6 (66.3; 92.0)	88.2 (66.3; 96.6)	0.847 (0.698; 0.941)	85.0
SSS	90.5 (69.6; 98.8)	73.7 (48.8; 90.9)	79.2 (63.9; 89.1)	87.5 (64.6; 96.4)	0.821 (0.667; 0.924)	82.5

mrTRG = modified MRI tumor regression grade; PPV = positive predictive value; NPV = negative predictive value; CI = confidence interval; SSS = split scar sign.

**Table 6:** Diagnostic performance of restaging MRI according to Reader to correctly identify near-complete response.

	Sensitivity, % (95%CI)	Specificity, % (95%CI)	PPV, % (95%CI)	NPV, % (95%CI)	AUC (95%CI)	Accuracy Rate, %
***Reader 1***						
T2WI score	83.3 (51.6; 97.9)	78.6 (59.0; 91.7)	62.5 (44.0; 78.0)	91.7 (75.4; 97.5)	0.810 (0.654; 0.916)	80.0
DWI score	75.0 (42.8; 94.5)	67.9 (47.6; 84.1)	50.0 (34.8; 65.2)	86.4 (69.7; 94.6)	0.714 (0.550; 0.846)	70.0
T1C score	75.0 (42.8; 94.5)	64.3 (44.1; 81.4)	47.4 (33.2; 62.0)	85.7 (68.4; 94.3)	0.696 (0.531; 0.832)	67.5
T2WI+DWI score	66.7 (34.9; 90.1)	71.4 (51.3; 86.8)	50.0 (33.0; 67.0)	83.3 (68.5; 92.0)	0.690 (0.525; 0.827)	70.0
T2WI+DWI+T1C score	66.7 (34.9; 90.1)	71.4 (51.3; 86.8)	50.0 (33.0; 67.0)	83.3 (68.5; 92.0)	0.690 (0.525; 0.827)	70.0
mrTRG	66.7 (34.9; 90.1)	71.43 (51.3; 86.8)	50.0 (33.0; 67.0)	83.3 (68.5; 92.0)	0.690 (0.525; 0.827)	70.0
***Reader 2***						
T2WI score	66.7 (34.9; 90.1)	85.7 (67.3; 96.0)	66.7 (42.6; 84.4)	85.7 (72.7; 93.1)	0.762 (0.601; 0.882)	80.0
DWI score	58.3 (27.7; 84.8)	92.9 (76.5; 99.1)	77.8 (45.9; 93.5)	83.9 (72.5; 91.1)	0.756 (0.595; 0.878)	82.5
T1C score	66.7 (34.9; 90.1)	85.7 (67.3; 96.0)	66.7 (42.6; 84.4)	85.7 (72.7; 93.1)	0.762 (0.601; 0.882)	80.0
T2WI+DWI score	66.7 (34.9; 90.1)	85.7 (67.3; 96.0)	66.7 (42.6; 84.4)	85.7 (72.7; 93.1)	0.762 (0.601; 0.882)	80.0
T2WI+DWI+T1C score	66.7 (34.9; 90.1)	85.7 (67.3; 96.0)	66.7 (42.6; 84.4)	85.7 (72.7; 93.1)	0.762 (0.601; 0.882)	80.0
mrTRG	66.7 (34.9; 90.1)	85.7 (67.3; 96.0)	66.7 (42.6; 84.4)	85.7 (72.7; 93.1)	0.762 (0.601; 0.882)	80.0

mrTRG = modified MRI tumor regression grade; PPV = positive predictive value; NPV = negative predictive value; CI = confidence interval.

**Table 7:** Interobserver agreement for restaging MRI in LARC after nCRT.

Reader 1—Reader 2	Kappa Cohen Coefficient of Agreement (SE)	*p*-Value
T2WI score	0.605 (0.102)	<0.001
DWI score	0.368 (0.102)	<0.001
T1C score	0.518 (0.103)	<0.001
T2WI+DWI score	0.544 (0.105)	<0.001
T2WI+DWI+T1C score	0.544 (0.105)	<0.001
mrTRG	0.544 (0.105)	<0.001
SSS	0.800 (0.093)	<0.001

mrTRG = modified MRI tumor regression grade; SSS = split scar sign; SE = standard error.

## Data Availability

The data presented in this study are available on request from the corresponding author. The data are not publicly available as written consent was not obtained from study participants for this.
